# Sydney Local Health District’s Integrated Response to the COVID-19 Pandemic: A Descriptive Study

**DOI:** 10.5334/ijic.5938

**Published:** 2022-09-30

**Authors:** Darith Liu, Hueiming Liu, Lou-Anne Blunden, Leena Gupta, Corey Moore, Sven Nilsson, Lisa Parcsi, Miranda Shaw, Teresa Anderson, John Eastwood

**Affiliations:** 1 Sydney Local Health District, Sydney, Australia

**Keywords:** Integrated Care, COVID-19, Pandemic, Sydney

## Abstract

**Introduction:**

Sydney Local Health District (SLHD) is a local health district in the state of New South Wales in Australia responsible for providing health services to the centre and inner west of the Sydney metropolitan area. SLHD adopted, during the COVID-19 pandemic, an integrated virtual and community care approach to manage quarantine and protect the health and wellbeing of the population.

**Description:**

The case study describes the roles of the different agencies and teams in the first six months of the pandemic across four key functions of 1) rapid screening and testing; 2) reaching the community; 3) effective quarantine and ongoing care; and 4) infrastructure, pathology and staff education.

**Discussion:**

The “whole of system” approach proved to be an effective method of delivering care that reduced community anxiety, improved and created relationships between existing and new internal and external stakeholders, and changed the community and health sector’s perspective on the importance of virtual care.

**Conclusion:**

This case study describes the importance of well-integrated, decentralised and funded public health system in response to the COVID-19 pandemic.

## Introduction

### Background and problem statement

The COVID-19 pandemic is an unprecedented international situation impacting individuals, communities and health systems. A strong population-based response is required to both halt the spread of the virus and to ensure coordinated health and social care of the population [1]. One study, utilising data on all-cause mortality, estimated the potential number of deaths in Australia to be over 16,000 if the country experienced similar COVID-19 outbreaks as in England and Wales [2]. By highlighting this scale of avoidable loss of life, the study argued for the importance of Australia’s pandemic response with respect to implementation of strategies, such as national border closure, early detection of cases, mandatory quarantine facilities, and public health measures such as hand hygiene and physical distancing advice.

Australia, as of 23 Nov 2020, has had 27,821 total cases, 907 deaths [3] out of the global situation of 58 million global cases, with 1.38 million global deaths [4]. In addition, 9 million COVID-19 tests have been conducted, with approximately a third being conducted in the state of New South Wales (NSW). As one of the health districts that contributed to the initial and ongoing screening, testing and quarantine of overseas travellers from Sydney International Airport, Sydney Local Health District’s (SLHD) response has played a significant role to the success of the Australian public health response to the pandemic.

SLHD was in a unique position, having prior to the pandemic established a virtual hospital (**rpa**virtual) that was integrated with a “whole of system” approach to management of population health and wellbeing. The “whole of system” approach built, in part, on other local “whole of system” integrated care initiatives, including Healthy Homes and Neighbourhoods and Living Well Living Longer Together with previous preparation and subsequent activation of a detailed local pandemic plan, this presented itself as an exemplar of a person-centred integrated care and population health approach. The scale and comprehensive nature of the SLHD pandemic response will be of interest to health administrators, policy makers, and clinical and scientific communities.

No one component described in this manuscript is itself innovative. The innovative nature of the initiative described here is the comprehensiveness of the “whole of system” approach. Studies of the complex and comprehensive nature of the pandemic response are not currently well documented. Stein and colleagues [1] argued for further health systems studies are required to inform and build the evidence base. The context and mechanisms at play in the SLHD pandemic response are analysed in more depth in an accompanying manuscript “Understanding for whom, how and why…” published in this journal [1].

This manuscript is part of a series of papers that describe, analyse and evaluate the integration, collaboration, outputs and outcomes of the SLHD integrated clinical and population health response to the COVID-19 pandemic. That programme of applied research used critical realist and pragmatic methodologies to evaluate the SLHD integrated response. The first of the critical realist studies [5], sought to understand for whom, how and why this SLHD response worked so as to inform a sustainable system transformation. Critical realist research is particularly focused on understanding how context and underlying mechanisms impact on outcomes. Future critical realist studies will examine the historical context impacting on integrated health and social care context within SLHD and NSW and will analyse in more detail some of the initiatives described in this paper and elsewhere [6,7,8,9].

The purpose of the case study presented here was to analyse the available operational documents in order to describe what the interventions were put in place, thus setting the “scene” for other related manuscripts, including: public health unit quality assurance [9], virtual hospital [7] and special health accommodation [6] initiatives.

The case study draws on a narrative synthesis of key documents, interviews and group discussions. The research questions for the case study were: 1) What did the different units at the SLHD do in response to the pandemic? 2) When were these measures implemented? 3) What were the numbers of community members screened and tested by each agency? 4) How did the roles and responsibilities of each of the units integrate and facilitate the SLHD response?

The narrative synthesis of key documents included analysis of 65 SLHD COVID-19 Operational Situation Reports [9]. Those reports were initially written daily, from May 2020, and later on a weekly basis. The reports provided an overview of key operational events, highlighting important data and trend within the local health district concerning the COVID-19 pandemic. Findings from the documentary analyses were discussed with the research team comprising of public health physicians, registrars and epidemiologists, and was also informed by twenty qualitative interviews with key managerial staff throughout the district.

### Ethical approval

Ethical approval was gained from the Human Research Ethics Committee (Royal Prince Alfred Hospital Zone) of the SLHD, X20-0310.

## Description of the integrated response

### Setting and Context

The state of New South Wales, Australia, is divided into 17 local health districts, and 2 Speciality Networks, which are responsible for managing all public hospitals and healthcare facilities for the population of a defined geographical area [10]. SLHD provides care to residents in central and inner west Sydney metropolitan area. Each local health district has a public health unit (PHU) responsible for protecting the community from communicable diseases or environmental harm within their geographical area and population. The pre-existing local relationships within this health system structure provides knowledge of the local population as well as easy communication channels between all the other PHUs, overseen by the NSW Ministry of Health.

The NSW Health Human Influenza Pandemic Plan was activated in response to the COVID-19 pandemic on 3 February 2020. The automatic activation of the SLHD local pandemic plan led to the establishment of the Flying Squad, the COVID-19 negative results hotline, “Tiger Teams”, redeployment of surge staff into relevant departments and facilities, and the implementation of Special Health Accommodation (SHA) for those requiring effective isolation and quarantine. Additionally, a newly established virtual hospital was able to quickly respond and provide care to COVID-19 patients in the community and in the SHA.

### Existing governance structure with a vision on integrated population and early pandemic preparedness

**Figure 1:**
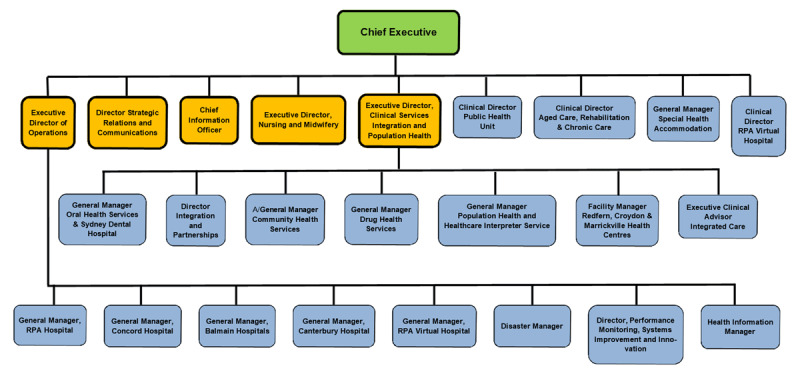
Governance and SLHD Context: The SLHD Senior Management Chart, as of May 2020. Only certain roles have been highlighted in the diagram; there are several more roles within the full organisational chart.

SLHD is an example of successful integration of different health units. The district comprises all public hospitals and healthcare facilities in central Sydney metropolitan area, overseeing Royal Prince Alfred Hospital (RPAH), Concord Hospital, Canterbury Hospital, Balmain Hospital, Sydney Dental Hospital and Oral Health Services, Community Health Services, Mental Health Services, Drug Health Services, Population Health, Public Health Unit, and **rpa**virtual. Figure 1 is an illustration of the district’s senior management chart.

The involvement of the SLHD Public Health Unit, Operations Team, Information and Communications Technology, Strategic Relations and Communications Team, and Community Health Services all played a key role in the response to the COVID-19 pandemic and the reduction of community transmission of the virus. The innovative services provided by SLHD in response to the COVID-19 pandemic to meet the needs of the community included **rpa**virtual, SHA, COVID-19 screening clinics and drive through clinics, community wellbeing clinics, and the rapid response (flying squad) teams. Table 1 provides an example number of tests and quarantined patients in the SLHD.

**Table 1:** The workflow of screening and testing of the community, responses to reach the community and quarantine, as of 20 July 2020 [11].


CATEGORY	DATE COMMENCED	CUMULATIVE TOTAL (20 JULY 2020)

**SCREENING CLINICS AND HOSPITAL PRESENTATIONS**		

Total COVID-19 screening presentation hospital & clinics	4 February 2020	46,551

Total COVID-19 tests hospitals & clinics	4 February 2020	39,361

Total COVID-19 screening presentation drive throughs	18 March 2020	66,536

Total COVID-19 tests drive throughs	18 March 2020	66,592

Total COVID-19 presentations for COVID-19 screening SLHD	4 February 2020	113,087

Total COVID-19 tests SLHD	18 March 2020	105,953

Total number of staff swabbed at drive through clinics	18 March 2020	1,768

**FLYING SQUAD**		

International Airport: number of people screened	4 April 2020	49,511

International Airport: number of people swabbed	4 April 2020	676

Domestic Airport: number of people screened	1 July 2020	3,091

Central train station: number of people screened	3 July 2020	1,583

**CORONAVIRUS NEGATIVE RESULTS HOTLINE**		

Completed cases in total	10 March 2020	92,910

**RPA VIRTUAL HOSPITAL CLINICAL CARE**		

Total individual **rpa**virtual COVID-19 positive patients	9 March 2020	720

Total individual **rpa**virtual COVID-19 negative patients	9 March 2020	787

**PUBLIC HEALTH UNIT**		

Total number of cases investigated and notified to the PHU	1 March 2020	99,470

Cumulative total of confirmed cases	1 March 2020	319

Number if suspected cases excluded by PHU	1 March 2020	99,151

Number of close contacts being followed up	1 March 2020	1465

**COMMUNITY WELLBEING CLINICS**		

Total number of people screened	15 April 2020	1144

Total number of tests	15 April 2020	142


The following sections are descriptions of the different roles across four key functions of: 1) rapid screening and testing; 2) reaching the community; 3) effective quarantine and ongoing care; and 4) infrastructure, pathology, and staff education.

## 1. Rapid screening and testing

### Public Health Unit

The SLHD Public Health Unit (PHU) is one of a number of public health units within the state, interacting with each other as well as other NSW Health departments, playing a critical role in protecting the community during the COVID-19 pandemic. The SLHD PHU interacted at the local level through their key tasks in case identification and investigations, contact tracing and contact management. This involved identifying new, suspected and probable COVID-19 cases, ensuring testing is carried out to confirm a case, contact tracing of those exposed to a confirmed case, isolating confirmed cases and quarantining their close contacts, ensuring cases and contacts have appropriate personal protective equipment such as masks, hand sanitisers and gloves, and ensuring the safe release of people from isolation once they were considered non-infectious.

The PHU normally consists of approximately 35 full time equivalent staff members, comprising doctors, nurses, environmental health officers, epidemiologists, and administrators. Assistance with the PHU’s pandemic response involved the redeployment of around 70 additional staff from within the district’s Population Health, Community Health and Planning teams to support contact tracing and administrative functions.

### Emergency Departments

Early preparedness to respond to the COVID-19 pandemic remained one of SLHD’s unique characteristics. The management of Emergency Department (ED) presentations was one clear example. Patients presenting to ED within the SLHD are all screened via an enhanced triage model based on the current definition of probable or suspected case according to the COVID-19 Communicable Diseases Network Australia (CDNA) National Guidelines [12]. The COVID-19 CDNA National Guidelines provide nationally consistent advice and guidance on best practice, according to the best available evidence, in response to COVID-19. Those who require emergency care and are identified as a suspect or probable case according to these guidelines are moved to an allocated area within the ED for further management. Patients only requiring assessment are then directed to the COVID-19 screening clinics for management and testing if required.

Patients tested in the ED or COVID-19 clinic were managed using the following system:

1. Hospital admission and monitoring of pending swab results if the patient is unwell.
2. Discharge home and monitoring of pending swab results if patient is well and able to self-isolate at home.
3. Discharge to temporary SLHD provided health accommodation and monitoring of pending swab results if the patient is well but unable to self-isolate effectively at home.

### Screening Clinics

Specific COVID-19 screening clinics provide screening, assessment, and testing of patients. These have reduced the presentations to ED in all hospitals within the district. The RPA COVID-19 clinic was the first coronavirus clinic to be established in the state, opening on 30 January 2020 as a “fever clinic.” RPAH receives most patients arriving straight from Sydney International Airport. As a result, the early establishment of a COVID-19 screening clinic was designed to treat this targeted population to further reduce potential risk to ED patients and staff. The very first patient identified as a suspect case arrived by ambulance from Sydney International Airport to be screened and tested for COVID-19 on 2 February 2020. Other clinics throughout the district were later established in March.

### Pop Up Clinics

SLHD established pop up COVID-19 testing clinics in areas of high vulnerability and housing estates, with assistance provided by community health staff, integrated care, engineering staff, flying squad and partner agencies such as the local city council and the local community groups. The district recognised geographically localised areas identified to have an elevated risk of community transmission of COVID-19, also known as “hotspots,” in which pop up clinics would be beneficial. SLHD also placed pop up clinics in geographical locations found to have low rates of COVID-19 testing to increase the testing rates within that community.

### Drive-Through Clinics

The COVID-19 drive through screening clinic commenced on the 17 March 2020 with services provided initially to the SLHD staff for screening, assessment and testing of COVID-19. The services later expanded to provide care to the general community, with further drive through clinics created in other areas within the district. The operation of one of the largest clinics, Summer Hill Clinic, was later handed over to Community Health Services with the assistance and support from the SLHD Emergency Operations Centre Team, SLHD Information, Communications and Technology Team, Tiger Teams and the Strategic Relations and Communications Team.

### Staff and Visitor Screening Stations

Staff and visitor screening stations were established at single points of entry into the hospitals and healthcare facilities within the SLHD at the beginning of March 2020. Being the first screening station in the state, other districts followed suit. COVID-19 screening questions about symptoms and travel history were asked and all individuals were monitored for fevers. Those identified to have risks (presence of symptoms, fever, or recent travelling history at a hotspot) would be directed to have COVID-19 testing and to self-quarantine at home. They were also advised to only return to work after a negative COVID-19 test result and resolution of their symptoms.

### Flying Squad

A rapid response team named the “flying squad” was organised by the SLHD Chief Executive on 9 March 2020 initially to perform screening at the Sydney International Airport. The team, initially consisting of experienced nursing and medical staff to collect nasopharyngeal swabs, later expanded to approximately 150 rostered staff. The team coordinated and assisted with screening and swabbing of patients on cruise ships, hospital accommodation, and in the community such as residential aged care facilities, boarding houses, train stations, pop up clinics, and drive through screening clinics. The flying squad is one example of a successful outreach service offered by the district to reduce hospital presentations and admissions and increased testing efficiency for the community.

## 2. Reaching the community

### COVID-19 Negative Results Hotline

The SLHD coronavirus hotline call centre (COVID-19 Negative Results Hotline) was established on 10 March 2020 and operated during the hours of 8AM to 10PM seven days a week. This telephone hotline was set up to contact patients with negative results to COVID-19 tests and to answer questions and concerns the patients in the community had in relation to their results, symptoms, and quarantine requirements. This service was unique as the only negative coronavirus hotline in the state. It contributed to reducing the community’s anxiety, allowed people to return to work earlier, and decreased phone calls made to places such as pathology laboratories, hospital switch board, and the public health unit. More than 50 staff members from the district were redeployed from Clinical Governance and Risk Unit, Community Health, Drug Health, Child and Family, and Information and Communication Technology teams to set up this special telephone hotline.

### Community Wellbeing Clinics

SLHD formed partnerships with organisations in the community in early April 2020 to protect and support populations with significant vulnerability during the COVID-19 pandemic. These included boarding house residents, people who are homeless, people living in social housing and people who speak limited English. There are 418 boarding houses in the district, accounting for 47 per cent of the registered boarding houses in NSW. Around 5,000 people currently reside in these boarding houses. The district collaborated with local councils, Land and Housing Corporation, Newtown Neighbourhood Centre, Riverwood Community Centre, Counterpoint and local general practitioners to establish a series of free pop-up community wellbeing clinics.

The services provided by these wellbeing clinics included screening and testing of COVID-19, influenza vaccinations, meals, and health and hygiene packs. A specialist allied health team, including social workers and mental health workers provided information and support for homeless people and boarding house residents to ensure their physical, emotional and social needs are met.

This integrated response to the pandemic is a strategy to reassure the community and increases awareness that their local health district cares for their health and social needs. By reaching out to the population who do not normally access health services, this approach aimed to reduce rates of influenza, reduce hospital presentations to the ED, and potentially reduce possible COVID-19 transmission in the boarding house and therefore reducing the risk of boarding house closures.

### Residential Aged Care Facility (RACF) Outreach Services

The district’s aged care clinical stream deserves a special mention for their extensive integrative involvement in the COVID-19 pandemic response. The elderly population are a vulnerable population in the community that is at increased risk of adverse outcomes from COVID-19. The pre-existing RACF outreach team that provides services to all aged care facilities within the district rapidly expanded to minimise the risk of a potential COVID-19 or influenza outbreak in this population. The Aged Care Outreach Team became one of the busiest teams in the geriatric clinical stream during the pandemic, visiting all of the 58 RACFs in the district. They screened and swabbed residents and staff members, with the assistance of the public health unit, microbiology, infection control and the flying squad. Additional services provided to the RACF include personal protective equipment (PPE) education, support with supply of PPE, infection control review and training.

## 3. Effective quarantine and ongoing care

### RPA Virtual Hospital

RPA Virtual Hospital (**rpa**virtual) is Australia’s first virtual hospital, launched in February 2020, to connect healthcare workers to patients remotely [7]. It was initially set up to support patients in the community across three different patient cohort types, none of which were COVID-19 related. This new model of care, which was coincidentally first operational on the same day as the state’s activation of the COVID-19 pandemic plan, rapidly pivoted to play in a critical role in the pandemic response in the management of positive COVID-19 cases requiring isolation in the community and quarantined travellers staying in the SHA.

**rpa**virtual developed a clinical model of care for COVID-19 positive patients, with automatic referral from the public health unit to the **rpa**virtual Care Centre. Initially, all positive patients received wearable devices to support remote monitoring of temperature and oxygen saturation rate, along with frequent clinical assessment via video-conferencing. Patients are now risk-stratified, with only high risk patients receiving wearables. All patients receive clinical assessment via video-conferencing and have access to the Care Centre 24 hours per day, 7 days per week.

In addition to providing clinical care to those in the community, **rpa**virtual was also tasked by the district’s Chief Executive to provide clinical governance for all patients and quarantined travellers in the SHA. This was met with a variety of challenges resulting in the development of new virtual models of care, and the deployment and recruitment of staff from a range of disciplines in order to adequately deliver high quality care to the patients in the SHA. For example, **rpa**virtual experienced a high number of pregnant women returning to Australia, some in the later stages of pregnancy without prior antenatal care. In response to this issue, a new virtual antenatal clinic was implemented, which required the deployment of a midwife clinical nurse consultant, midwives, and regular collaboration with the obstetrics and gynaecology medical team.

A surge of ex-patriots returning back to Australia after living overseas was also observed, presumably due to the protection provided by our public health response and access to our universal healthcare and social support services. The complex health care needs of this largely geriatric population within the SHA resulted in the rapid need for a new model of care involving the introduction of regular virtual geriatric ward rounds to perform falls assessments, cognitive assessments, and mobility assessments.

All virtual models of care were developed in close consultation with the relevant specialist department, with agreed and documented clinical protocols, and escalation and referral pathways. This was particularly important to minimise the movement of patients outside of hotel quarantine.

The services provided by **rpa**virtual demonstrated that some healthcare, which has been traditionally treated within hospitals, could be delivered safely using technology in the community and in SHA. **rpa**virtual reduced the number of hospitalisations and presentations to the district’s EDs. The establishment of **rpa**virtual initially met with some scepticism and resistance pre-pandemic. Subsequently **rpa**virtual has now gained wide acceptance within the district and state-wide with the winning of the NSW Government Premier’s Award in Excellence in Digital Innovation.

### Special Health Accommodation (SHA)

The district provided accommodation for members of the community who were either COVID-19 positive or were identified as close contacts, and were unable to effectively self-quarantine or self-isolate at home due to their personal living arrangements and health needs. Those services were later extended to all returned overseas travellers on 31 March 2020 after the NSW Public Health Order (COVID-19 Air Transportation Quarantine and COVID-19 Maritime Quarantine) was enacted that mandated that all people entering Australia must quarantine in hotels or a healthcare facility.

The SLHD SHA was the first Australian “health hotel” for isolation, quarantine and clinical management [6]. The intervention served not only as part of the local district’s response to the pandemic, but also contributes to the state’s response, with connections facilitated by information and communications technology. The SHA functioned as a 650 subacute hospital with a similar governance structure being overseen by a General Manager, Director of Nursing and Clinical Director. Approximately 50 registered and assistant nurses conduct twice daily welfare checks and continuous 24 hours a day clinical care. Further support is provided by allied health workers, including occupational therapists, dieticians, and social workers. There is also an escalation process to a psychologist or a physician if an increased level of care is required. All COVID-19 patients in the SHA also receive care from **rpa**virtual and are transferred immediately to RPA hospital if they become unwell.

In response to the COVID-19 CDNA National Guidelines, mandatory day 10 and day 13 exit swabs were initiated with the assistance of the Public Health Unit, **rpa**virtual, flying squad and microbiology department. In addition, each individual’s circumstances were reviewed prior to discharge by the **rpa**virtual discharge team, public health unit and Chief Executive to ensure a non-infectious state and fulfilment of the quarantine. These measures likely contributed to the containment of COVID-19 in NSW during 2020.

After the closure of Australia’s borders on 20 March 2020, there was a gradual decrease in the number of COVID-19 cases, which further decreased after the introduction of mandatory hotel quarantine. Major outbreaks that have occurred in Australia since the initial wave of infections have been all linked to quarantine hotels [13]. This highlights the importance of strict infection control in hotel quarantine. To date, no COVID-19 outbreaks have been linked to cases managed in the SLHD SHA.

## 4. Infrastructure, pathology, and staff education

### Information and Communications Technology (ICT)

The SLHD ICT delivered an extensive amount of work as part of the pandemic response, overseeing all communications within the district for over 250 projects. In late 2019, ICT worked closely with the **rpa**virtual team to establish the technology infrastructure required to launch the new Care Centre and then to source necessary wearable devices for remote monitoring. Other services provided by this group included telehealth support, particularly for SHA and aged care, enabling working from home and remote access, rapid response for Zoom meetings, hospital visitor management systems, and patient portals for screening and drive through clinics.

A common results portal, titled “Are My Results Ready?” across all COVID-19 screening clinics and facilities was established to ensure that results were all streamlined within the local health district. This project enabled patients to access their negative results online. This portal also provided reports to the SLHD COVID-19 Hotline so patients who have not yet received their results were prioritised to be contacted first.

### NSW Health Pathology

At the beginning of the pandemic, PCR tests were sent to one of the two specialist virology laboratories within NSW. In March 2020, NSW Health Pathology began performing COVID-19 PCR testing at RPA to meet increasing testing demands. It now performs the bulk of testing for the state, largely due to the testing requirements of the occupants in the SHA.

As part of an initiative by the district’s Chief Executive, the NSW Pathology SMS Service was implemented to provide prompt results to those in the community who were tested negative. Rapid testing and results allowed the community and staff to quickly return to their work. The introduction of this SMS service also allowed the COVID-19 negative results hotline to prioritise patients to call and reduced the workload of the staff at the hotline.

### Tiger Team

A Tiger Team was established as a pandemic innovation to provide care and support for front line staff throughout the COVID-19 response. Observing the challenges faced by healthcare workers overseas presented an opportunity for the District to plan a strategy to avoid similar events locally. The members of the Tiger Team were assigned to every ward in the district’s facilities to deliver education on COVID-19, infection control, and personal protective equipment, as well as ensuring staff welfare. The low rate of COVID-19 cases and transmission to healthcare staff in the hospitals, and particularly in the health hotels serves as one demonstration of the Tiger Team’s achievement in keeping healthcare workers safe.

Another accomplishment was the mask fit testing pilot programme as a response of the PPE shortage, which initially presented a huge area of anxiety among all frontline staff members. The Tiger Team, along with the district’s PPE governance committee, which comprised of the executive director of operations, infection control, infectious diseases, anaesthetists and other clinical leads, have worked collaboratively on a programme to ensure each staff member has had a mask fitting test. This pilot later became a policy with the state’s Ministry of Health and later enacted in other local health districts.

### Strategic Relations and Communications

Prompt delivery of communication directly impacting staff was made possible with the assistance of the district’s Strategic Relations and Communications team. This included messages from the Chief Executive, staff information sessions, and electronic distributed messages (EDMs). It was within this team’s responsibilities to ensure all information within each of the district’s facilities were up to date and consistent with the NSW Health website. Education of staff was also a major task throughout the pandemic, particularly in the delivery of education and training content such as PPE donning and doffing posters and hand hygiene posters. The team not only supported the design of those in collaboration with Infection Control, Infectious Diseases and the Public Health Unit, but also printed them, laminated them, and helped facilitate the Tiger Teams in the distribution of these posters to all facilities.

## Discussion

Stein and colleagues [1] note that COVID-19 has thrown into sharp relief the problems that fragmented health and care systems face in adapting to crises that require an urgent and collaborative response. The SLHD is accountable and responsible through legislation and funding agreements for the health and well-being of all people residing within a defined geographical area. Advanced “whole of system” planning and attention to meeting the needs of vulnerable populations put SLHD in a good position to respond to the COVID-19 pandemic while at the same time contributing to air and seaport border quarantine requirements in Australia’s largest international port of entry.

As noted in the introduction, SLHD was in a unique position, having prior to the pandemic established a virtual that was integrated with a “whole of system” approach to management of population health and wellbeing. We have previously reported on the SLHD development of a person-centred integrated care policy framework [14]. That “whole of system” approach built, in part, on other local “whole of system”, person-centred integrated care and population health approaches [14,15,16,17].

Those pre-COVID-19 processes led to the establishment of “whole of health” and “whole of system” governance structures, and a range of hospital avoidance initiative including the establishment of **rpa**virtual in late 2019 prior to the pandemic. The nature and extent of those initiatives is beyond the scope of this paper. Taken together, however, they illustrate a commitment by SLHD to effective and participatory leadership across the health and social care system.

Effective and participatory leadership, robust and flexible finances, ability to redistribute resources, and flexibility in the delivery of care are key features of health system resilience [18]. Leadership played an important role in the development of the culture for the SLHD response. The importance of leadership was highlighted recently by Eastwood and Miller [19] citing the work of Evans and colleagues [20]. They note that “System leaders at a senior level will often work through partnership structures and management networks, whereas system leaders at a clinical level will work through care pathways, multi-disciplinary teams, and professional networks. Both will need a similar set of skills, values and facilitative style of leadership” [20].

A top-down structure is essential in a public health response to the pandemic, as was initially outlined and planned for in the epidemic preparedness plan, which allowed for the effective response. The participatory leadership, however, also enabled nimble adaptations to local problems within and across each agency. For example, this included the close working relationship between public health unit, **rpa**virtual and the special health accommodation.

During the pandemic, the style of leadership moved from one of participatory leadership toward “Incident Command”. We note in accompanying qualitative study [5] that “many informants described how the existing … governance structures facilitated the adaptation to the “incident command” strategy .. [where] the Chief Executive became ‘commander, oversight person’ who was strongly supported by a network of clinicians and managerial staff”. In the SLHD context the “whole of health” and “whole of system” interagency relationships were also important.

SLHD’s response to the COVID-19 pandemic has demonstrated that internal and external stakeholders can rapidly and efficiently work together to produce a robust integrated population and public health response taking account of clinical, social, cultural and other needs in a way that is proportionate, evidence-based and equitable. This saw the district adopt a coordinated population health approach that changed the traditional focus on hospital sector and specialist care to integrated community care. From this approach, the COVID-19 pandemic has provided an opportunity for the SLHD to transform to an effective delivery of care that is able to reduce community anxiety, improve relationships with existing and new partners among internal and external stakeholders, and change the community and health sector’s perspective on the importance of virtual care and telehealth.

Existing governance structure of the SLHD allowed for the early establishment of the whole of local health district steering committee led by the Chief Executive as incident controller.

The SLHD’s focus on the community and population health presented a resilient system for responding to the pandemic. This allowed prompt screening and testing for COVID-19, the ability to prioritise testing equipment availability, and to have proactive detection in the community. The utilisation of the Flying Squad, for example, to test suspected cases at the airports, train stations, aged care facilities, drive through clinics, and community wellbeing clinics contributed to the early detection of cases and subsequently reduced avoidable ED presentations and hospitalisations. Services in the SLHD were stood up early and there was capacity to surge to support a state-wide quarantine health hotel.

Moreover, the COVID-19 pandemic acted as a catalyst in providing an opportunity for the district’s staff to embrace community care led strategies to respond to the pandemic. In addition, COVID-19 reinforced and strengthened the importance of **rpa**virtual and community health. For instance, there was elevated motivation for the district’s staff to adopt technology they would normally be resistant to, increasing the value of the services provided by ICT, and providing the proof of concept and the value of **rpa**virtual. The urgency at which the district had to respond motivated different facilities and departments working with a common vision to develop creative innovations to adapt to the pandemic. Indeed, staff reported how an initial reticence towards telehealth and virtual care was overcome, after seeing how effective virtual care is. The pandemic forced the district’s staff to rethink existing strategies, adapt to the delivery of healthcare through telehealth, and appreciate the importance of workforce flexibility.

The focus on reaching the community such as targeted pop up clinics, community wellbeing clinics, and the integrated strategy for supporting vulnerable and homeless communities, highlight that a coordinated response can prevent exacerbating inequalities in care. Indeed, the second surge of COVID-19 cases through the housing dormitories in Singapore provided valuable lessons on the importance of addressing the social determinants of health. The second wave in Melbourne and the initial problems in residential aged care facilities demonstrated the relevance of inter-sectoral integration between health, housing, police and city councils, especially in high density and vulnerable populations. Other similar examples also described Singapore’s resilient health care system during the COVID-19 pandemic, with the country developing a whole-of-nation approach implemented as a result of lessons learnt from the 2003 SARS outbreak [21]. Multiple ministries within the government worked closely and collaboratively with clinical and non-clinical organisations to lead and implement measures to protect their citizens.

Finally, SLHD maintained ongoing clear communication channels with the NSW Health departments and other agencies involved in the nation and state-wide pandemic plan. The district’s vision for a whole of system population health approach was vital in response to the pandemic. Supportive leadership and strong governance structure, while maintaining the flexibility to absorb and adapt to the pandemic enabled a successful integrated response across agencies such as public health units, **rpa**virtual, community and population services to rapidly screen and test, effectively quarantine and reach the community and address the needs of the vulnerable populations; while continuing with maintaining core health service capabilities.

This descriptive study of the SLHD integrated response to the COVID-19 pandemic serves as a building block for a larger mixed methods study that will utilise qualitative data from semi-structured interviews from key informants in the decision making of the pandemic response. The mixed methods study will further our understanding of integration measurement and outcomes that can be applied for future similar pandemics or other outbreaks of this pandemic.

## Conclusion

The SLHD’s initial response to the COVID-19 pandemic was comprehensive and contained many unique features. It is through the several challenges faced by this district, together with a strong leadership and governance structure, good communication between all levels, and the existing health care system in NSW that enabled the adaptation to successful innovative services. This contributed to effective containment, which likely reduced community transmission of COVID-19.

Our main learning points include that the culture of innovation, meant that we could quickly adapt a telehealth model of care to support people with COVID-19 in the community and in the special health accommodation; that we had an adaptive and willing workforce that could be trained to work as speciality teams and deployed quickly. Challenges include overlapping jurisdictions, and lack of standardisation of processes, that needed to be overcome quickly through respectful and clear lines of communication as SLHD had an epidemic preparedness plan with established leadership and governance structure.
